# Does *Pseudomonas aeruginosa* Colonization Affect Exercise Capacity in CF?

**DOI:** 10.1155/2019/3786245

**Published:** 2019-12-09

**Authors:** Asterios Kampouras, Elpis Hatziagorou, Vasiliki Avramidou, Vasiliki Georgopoulou, Fotios Kirvassilis, John Tsanakas

**Affiliations:** ^1^Pediatric Pulmonology and CF Unit, 3^rd^ Department of Pediatrics, Hippokration Hospital, Aristotle University of Thessaloniki, Thessaloniki, Greece; ^2^Radiology Department, Hippokration Hospital, Thessaloniki, Greece

## Abstract

**Introduction:**

Cardio-Pulmonary Exercise Testing (CPET) has been recognized as a valuable method in assessing disease burden and exercise capacity among CF patients.

**Aim:**

To evaluate whether *Pseudomonas aeruginosa* colonization status affects Exercise Capacity, LCI and High-Resolution Computed Tomography (HRCT) indices among patients with CF; to check if *Pseudomonas* colonization can predict exercise intolerance.

**Subjects:**

Seventy-eight (78) children and adults with CF (31 males) mean (range) age 17.08 (6.75; 24.25) performed spirometry, Multiple Breath Washout (MBW) and CPET along with HRCT on the same day during their admission or follow up visit.

**Results:**

78 CF patients (mean FEV1: 83.3% mean LCI: 10.9 and mean VO_2_ peak: 79.1%) were evaluated: 33 were chronically colonized with *Pseudomonas aeruginosa*, 24 were intermittently colonized whereas 21 were *Pseudomonas* free. Statistically significant differences were observed among the three groups in: peak oxygen uptake % predicted (VO_2_ peak% (*p* < 0.001), LCI (*p* < 0.001), as well as FEV1% (*p* < 0.001) and FVC% (*p* < 0.001). *Pseudomonas* colonization could predict VO_2_ peak% (*p* < 0.001, *r*^2^: −0.395).

**Conclusion:**

Exercise capacity as reflected by peak oxygen uptake is reduced in *Pseudomonas* colonized patients and reflects lung structural damages as shown on HRCT. *Pseudomonas* colonization could predict exercise limitation among CF patients.

## 1. Introduction

Cystic Fibrosis lung disease is characterized by the presence and persistence of thick mucus secretions that are not easily cleared from the patient's airways [1, 2]. This decreased mucus clearance predisposes to early colonization from pathogens, especially *Pseudomonas aeruginosa (P. aeruginosa),* thus leading to a vicious cycle of infection and inflammation [3]. *Pseudomonas* colonization has been shown to be a factor influencing FEV1 decline in cystic fibrosis, increasing the hazard of developing severe lung disease to 2.4 times [4] and consequently having a strong impact on overall prognosis.

Since many years, prognosis in CF is best evaluated with Cardiopulmonary Exercise Testing (CPET) [5, 6]. Exercise testing measures the peak oxygen volume absorbed (VO_2_ peak)by a person's lungs during maximal exercise highlighting his exercise capacity. In CF, patients with VO_2_ peak values >80% predicted have been shown to present nearly excellent 10-year prognosis [5]. *Pseudomonas* colonization as a factor of inflammation and infection has been shown to affect exercise capacity. Recently, Van de Weert-van Leeuwen et al., have showed that *Pseudomonas* is an independent factor associated with longitudinal changes in VO_2_ peak and exercise capacity.

Even though long-term effects of *P. aeruginosa *colonization in VO_2_ peak have been made profound, no data exist on how exercise capacity of CF patients colonized compares to that of *Pseudomonas*-free and if such impairment exists, whether it is translated to structural lung damages as seen on High Resolution Computed Tomography (HRCT).

Aim of the present study was to perform a cross-sectional analysis of exercise capacity in CF patients chronically colonized with *P. aeruginosa*, intermittently colonized or free of any pathogen, and also to investigate possible differences in HRCT and whether any correlations with exercise capacity exist.

## 2. Materials and Methods

A total of 78 CF patients mean age 14.9 years old (4.69) attending the CF center of our Department, participated our study. Of these, 73 (93.6%) were on pancreatic replacement therapy. Intermittently colonized with *P. aeruginosa *were 24 (30.8%), 33 (42.3%) of them were chronically colonized while 21 were *P. aeruginosa* free.

### 2.1. Anthropometry

Height and weight were measured in light clothing. Anthropometric data are shown in Table 1.

### 2.2. Spirometry

Standard spirometry with an electronic spirometer (Vitalograph 2120, Vitalograph Ltd., Ennis, Ireland) according to the ATS/ERS standards [7] was applied in order to estimate Forced Vital Capacity (FVC) and forced expiratory volume in one second (FEV1). Data were expressed in %predicted using the normative data from the Global Lung Function Initiative software (GLI 2012, Global Lung Function Initiative Task Force, available at: http://www.lungfunction.org/).

### 2.3. Multiple Breath Washout

MBW measurements were performed with a flow, volume and molecular measurement analyzer (EXHALYZER D, Ecomedics, Switzerland), according to ERS/ATS Consensus Guidelines [8]. LCI is defined as the number of FRC lung turnovers (TO: Cumulated Expired Volume divided by the Functional Residual Capacity (FRC)) required to reduce end-tidal N_2_ concentration to 1/40 of the starting concentration, which accounts to 2.5% of the initial one. A pulmonary disease resulting in uneven ventilation distribution, prolongs the duration of the washout, thus elevate the LCI. Normal LCI values are found to be lower than 7 [9, 10]. Moment ratio M1/M0 and M2/M0 emphasize on delayed gas washout due to peripheral airway regions pathology [11]. Ventilation distribution is performed through convection and diffusion. Two other indices have been proposed to express pathology throughout the trancheobrocheal tree: (a) Scond, which represents convection-dependent inhomogeneity and (b) Sacin that represents diffusion convection-interaction-dependent inhomogeneity [8]. Finally, FRC expresses the remaining air in the lungs at the end of expiration which is in direct relevance with the airway opening [12, 13].

### 2.4. Cardiopulmonary Exercise Testing (CPET)

All participants performed a maximal cardiopulmonary exercise testing on a cycle ergometer (Ergoline, Vmax Series V.20-1, Sensor medics). Cardiac parameters were also measured (cardiograph model Corina, S.N. 101164361, Cardiosoft software V5.15). A Gofrey protocol for exercise testing was applied [14]; according to that, depending on subject's height, after baseline measurements for 1 minute and a warm-up period of 2 minutes cycling with 10 watts (for patients <120 cm tall), 15 watts (120–150 cm) or 20 watts (>150 cm tall),work load was increased by 10, 15, 20 watts respectively every minute until volitional fatigue. Exercise time was kept within 8–12 min. Patient's heart rate over 85% of maximum predicted [15] along with Respiratory equivalence ratio (RER) over 1.05 [16] were used as indicators of a maximal test. The following parameters were measured: peak oxygen uptake (VO_2_ peak), peak oxygen uptake/weight (VO_2_ peak/kg) ventilator equivalent ratios for oxygen and carbon dioxide at peak exercise (VE/VO_2_, VE/VCO_2_), anaerobic threshold (AT), breathing reserve at peak exercise (BR%), dead space to exhaled volume ratio (Vd/Vt). VO_2_ peak% predicted was calculated using the Orenstein gender specific equations [17]:

(1) $$\begin{array}{c} \text{Girls}:{\text{VO}}_{2}\;\max\;\left(\;\text{l}/\min\right)=0,0308806\times \text{Height}\;\left(\text{cm}\right)-2,877, \end{array}$$

(2) $$\begin{array}{c} \text{Boys}:{\text{VO}}_{2}\max\;\left(\text{l}/\min\right)=0,044955\times \text{Height}\;\left(\text{cm}\right)-4,64. \end{array}$$

Then the VO_2_ peak% predicted was calculated.

Breathing reserve was calculated as: MVV−VE/MVV (MVV = maximal voluntary ventilation; (MVV = 35 × FEV1); VE = maximum exercise ventilation). Anaerobic threshold (AT) was determined by the Sensor Medics software, using the VCO_2_/VO_2_ plot.

### 2.5. HRCT

HRCT scans were performed on an (Asteion) Toshiba CT scanner. Slices measuring 1.5 mm were obtained at 10 mm intervals during suspended respiration in supine position. Additional expiratory scans were obtained in older cooperative children at 20 mm intervals. Each CT scan was scored using the “Bhalla” scoring system by an experienced radiologist; the Bhalla score shows high inter-observer reproducibility and sensitivity [18, 19]. The following changes were evaluated: severity and extent of bronchiectasis, severity of peri-bronchial thickening, generation of bronchial division involved, extent of mucus plugging, sacculation or abscess formation, bullae, emphysema, atelectasis and consolidation. Higher values of the Bhalla score indicate more severe lung disease.

### 2.6. Statistical Analysis

Descriptive statistics were used to describe study population. All parameters were described as mean and standard deviation (sd). Kolmogorov–Smirnov test was applied to express normality while Spearman's Correlation Coefficients were used to assess for possible correlations between above parameters. Correlation coefficient values <0.10 represent weak correlation, 0.20 weak to moderate, 0.25–0.35 moderate correlation, 0.40 moderate to strong whereas values >0.50 were indicative of strong correlation [20].

## 3. Results

In total 78 CF patients mean age (sd) 14.9 (4.68) years participated. Mean FEV1 (%predicted) was 87.5%, mean LCI 10.87 and mean VO_2_ peak% predicted was 79.1%. Of all patients, intermittently colonized with *P. aeruginosa* were 24 (30.8%), 33 (42.3%) of them were chronically colonized while 21 were *P. aeruginosa* free.

**Figure 1:**
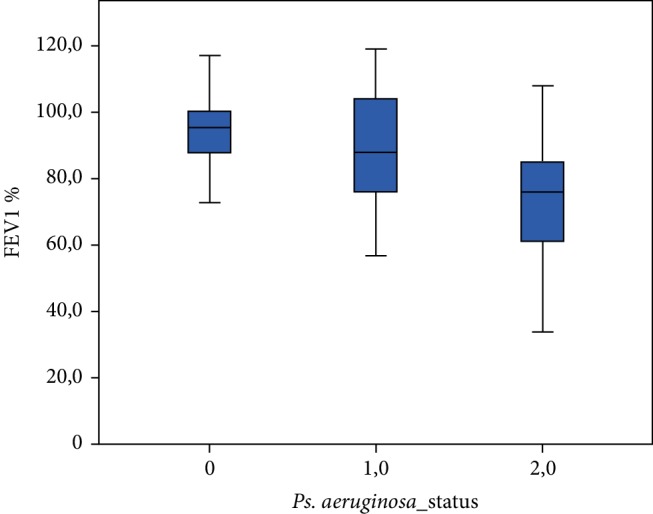
FEV1% predicted among the three groups of patients. (0: patients not colonized, 1: patients intermittently colonized with *P. aeruginosa*, 2: patients chronically colonized with *P. aeruginosa*).

**Figure 2:**
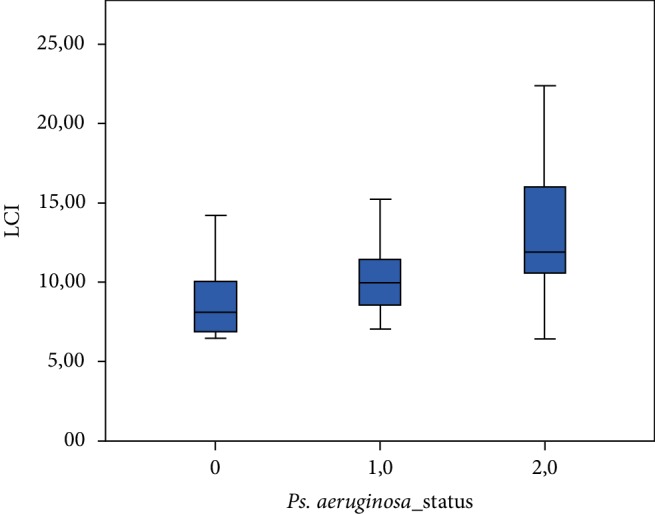
LCI among the three groups of patients. (0: patients not colonized, 1: patients intermittently colonized with *P. aeruginosa*, 2: patients chronically colonized with *P. aeruginosa*).

**Figure 3:**
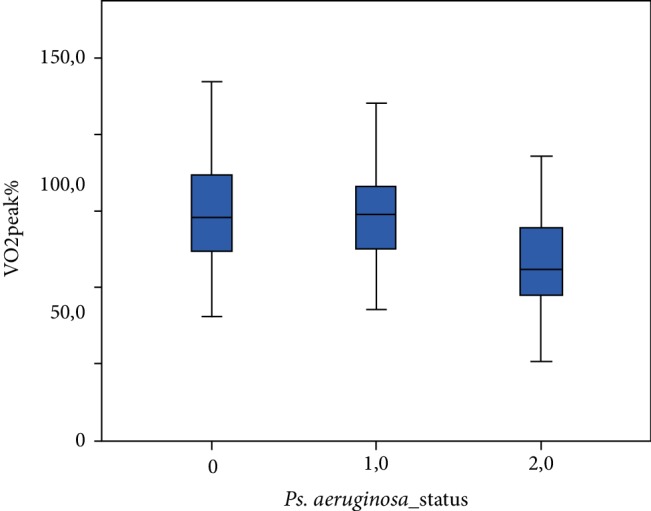
VO_2_ peak% predicted among patients not colonized, intermittently and chronically colonized with *Pseudomonas aeruginosa*. (0: patients not colonized, 1: patients intermittently colonized with *P. aeruginosa*, 2: patients chronically colonized with *P. aeruginosa*).

**Figure 4:**
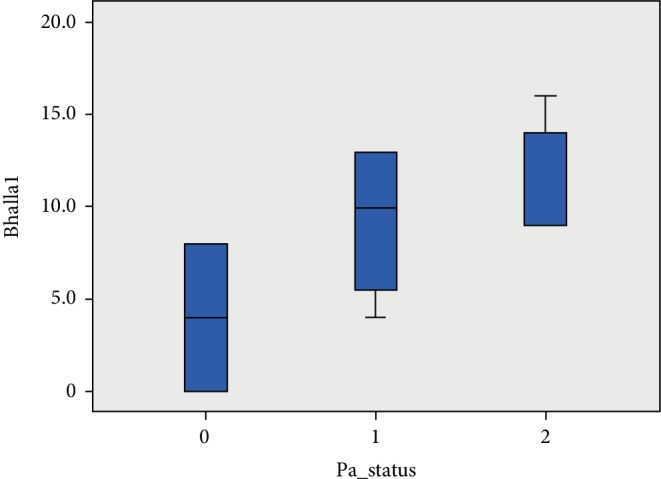
Bhalla scores among patients not colonized, intermittently and chronically colonized with *Pseudomonas aeruginosa*. (0: patients not colonized, 1: patients intermittently colonized with *P. aeruginosa*, 2: patients chronically colonized with *P. aeruginosa*.

Spirometric, MBW, CPET and Bhalla score values among the three patient groups are shown in Table 2. FEV1 (%predicted) differed significantly (*p* ≤ 0.001) between the three groups (Figure 1). There was a significant difference noted for the LCI (Figure 2), while patients chronically, intermittently and not colonized with *Pseudomonas aeruginosa* presented significant differences concerning their VO_2_ peak. (*p* < 0.001) (Figures 3 and 4).

When regression analysis was applied, *Pseudomonas* status was shown to be a strong predictor of exercise limitation in CF patients (*p*<0.0001, *r*^2^: −0.395).

## 4. Discussion

The main finding of this study is that *P. aeruginosa *colonization is always indicative of exercise intolerance and thus impaired activity in CF even if the patient's respiratory function is preserved.

During the past years progress in the treatment of Cystic Fibrosis has led to an overall increase in the expected life span for CF patients [21]. This improved prognosis has shifted attention to improving quality of life especially in the adolescent and adult CF population. Major determinant of quality of life is exercise capacity-the maximum physical exertion that a person can sustain.

Exercise capacity is at best evaluated with a full Cardiopulmonary Exercise Test (CPET). Peak oxygen uptake upon maximal exercise (VO_2_ peak) has been shown to be a significant predictor of life expectancy not only in CF patients but also in healthy population [22]. Even though CPET can provide valuable information regarding a patient's status, few CF centers worldwide implement exercise testing as a part of their routine CF evaluation [23, 24].

In the present study, we showed that patients colonized with *P. aeruginosa *have impaired exercise capacity and Lung Clearance index in contrast to noncolonized. Apart from that, it was also shown that chronic colonization affects exercise capacity even more than intermittent colonization and can predict structural lung damages, along with functional capacity of CF patients.

Chronic *P. aeruginosa *infection leads to a vicious cycle of inflammation and subsequent airway remodeling [25, 26]. These structural lung damages in CF patients lead to impaired gas exchange [27]. Even though this pathway to impaired gas exchange has been nearly clarified, no studies were performed before trying to look into the causality effect of *Pseudomonas* status on HRCT abnormalities. In our study, *P. aeruginosa *colonization was proven to be a prognostic factor of structural lung damages and an index of impaired exercise capacity. Thus, our study fills a gap in existing literature highlighting the fact that *Pseudomonas* can be an indicator of impaired exercise capacity which can be followed by alterations in HRCT.

Our finding comes in accordance to the findings of Van de Weert-van Leeuwen et al., who showed that longitudinally, *Pseudomonas* colonization can affect exercise capacity. The fact that *Pseudomonas* colonization is indicative of impaired exercise capacity and thus an additional burden in everyday activities of CF patients can be helpful to CF centers that are not able to perform exercise testing. Physicians with patients colonized intermittently or chronically could use this knowledge to prescribe exercise rehabilitation even in an early stage in order for daily activities to not be hassled [17].

A few limitations should be taken into consideration though. Even though our study looks into VO_2_ peak and Bhalla score in patients with preserved respiratory function colonized or not, differences in FEV1% predicted and LCI between groups were significant. Having three groups with similar respiratory function would lead to safer results. However, as *Pseudomonas* status affects respiratory function in CF this could not be possible in our study nor the adjustment for it. Perhaps a study with larger cohorts could clarify this. Additionally, even though the effects of *P. aeruginosa *colonization on exercise capacity seem obvious, no studies before have looked into the extent of this effect nor correlate it with structural damages as shown on HRCT.

In conclusion, *Pseudomonas aeruginosa* colonization in CF youths is well associated with structural lung damages and impaired exercise capacity. Actions to improve activity status should be taken as soon as colonization is known.

## Figures and Tables

**Table 1:** Demographical characteristics of patients participating in the study.

	Median (sd)/*N* (%)
Patients	78 (31 males)
Age	14.90 (4.68)
Weight (kg)	47.17 (13.44)
Height (cm)	153.66 (14.34)
BMI (kg/m^2^)	19.57 (3.32)
ΔF508 homozygous/heterozygous	20 (25.6%)/38 (48.7%)
Pancreatic sufficient (%)	73 (93.6%)
PsA colonized	
Intermittently	24 (30.8%)
Chronically	33 (42.3%)

**Table 2:** Spirometric, MBW, CPET and Bhalla score values in the 3 patient groups (values are shown as mean (StDev)).

	Free of *P. aeruginosa*	Intermittently colonized	Chronically colonized
FEV1% pred	108.0 (13.9)	91.6 (18.4)	73.1 (21.0)
FVC% pred	107.0 (17.5)	97.0 (17.9)	80.3 (18.7)
FEF_50_% pred	100.5 (16.9)	81.2 (30.8)	61.8 (30.9)
LCI	8.6 (2.2)	10.4 (2.5)	13.3 (4.0)
Bhalla score	4 (5.65)	5.5 (2.38)	11.00 (4.2)
VO_2_ peak%	91.3 (26.3)	83.5 (24.0)	68.5 (18.3)

## Data Availability

The data used to support the findings of this study were supplied by Elpis Hatziagorou. Requests for access to these data should be made to [Elpis Hatziagorou, ehatziagorou@gmail.com].
